# The change of retinal microvascular in APAC eyes and fellow PACS eyes detected using wide-field swept-source optical coherence tomographic angiography

**DOI:** 10.3389/fmed.2025.1618436

**Published:** 2025-07-02

**Authors:** Rumin Zhao, Wenhui Geng, Zijian Zhang, Yunlong Wu, Xiaotong Wang, Bojun Zhao

**Affiliations:** ^1^Department of Glaucoma, Shandong Lunan Eye Hospital, Linyi, China; ^2^Department of Ophthalmology, Shandong Provincial Hospital Affiliated to Shandong First Medical University, Jinan, China

**Keywords:** acute primary angle closure, primary angle-closure suspect, wide-field OCTA, superficial vascular complex, retinal nerve fiber layer

## Abstract

**Purpose:**

This study aimed to quantitatively evaluate changes in vascular parameters in a 12 mm × 12 mm region centered on the fovea with wide-field swept-source optical coherence tomographic angiography (WF SS-OCTA), and establish their correlations with structural parameters in acute primary angle closure (APAC) eyes, as well as in fellow primary angle-closure suspect (PACS) eyes.

**Methods:**

In this retrospective study, WF SS-OCTA was utilized to measure vascular parameters in a 12 mm × 12 mm region centered on the fovea in 31 patients (31 APAC eyes and 31 fellow PACS eyes). Vascular parameters included vessel density (VD) of the superficial vascular complex (SVC) and deep vascular complex (DVC). Structural parameters comprised macular ganglion cell complex (GCC) thickness and peripapillary retinal nerve fiber layer (pRNFL) thickness. Pre- and postoperative (3 month after APAC attack) differences between APAC and PACS eyes were statistically analyzed.

**Results:**

No significant differences were observed in vascular density or structural parameters in PACS eyes pre- and postoperatively (*p* > 0.05). In APAC eyes, postoperative SVC VD showed no significant change in the 0–6 mm region but decreased significantly in the 6–12 mm annular region centered on the fovea (*p* < 0.05). DVC VD increased across all scanned regions postoperatively (*p* < 0.05). The average pRNFL thickness, quadrant-specific pRNFL thickness, and GCC thickness were significantly reduced after operation (*p* < 0.05). Multivariate linear regression revealed positive correlations between GCC thickness, SVC VD (0–6 mm, 0–12 mm, 6–12 mm), and pRNFL thickness (*p* < 0.001).

**Conclusion:**

Wide-field SS-OCTA revealed spatially distinct vascular responses in APAC eyes: underlying axonal loss with concomitant SVC compromise in the 6–12 mm annular region centered on the fovea and partial DVC recovery at 3 months after APAC attack, highlighting WF SS-OCTA’s utility in monitoring APAC progression.

## Introduction

Glaucoma is one of the leading causes of irreversible blindness worldwide, and it is proposed that the number of glaucoma patients globally will rise to 111.8 million by 2040 (1). Glaucoma is a progressive, multifactorial optic neuropathy characterized by the progressive death of retinal ganglion cells (RGCs) and loss of axons, thinning of the retinal nerve fiber layer (RNFL), and ultimately leading to visual field defects or even blindness (2, 3). Primary angle-closure disease (PACD) is the most common type of glaucoma in China (2, 4). Acute primary angle closure (APAC), a special form of PACD as classified by the International Society of Geographical and Epidemiological Ophthalmology (ISGEO) staging system (5), is an ophthalmic emergency. Its primary feature is the sudden closure of the anterior chamber angle, resulting in a sharp increase in intraocular pressure (IOP). If not treated promptly, APAC can severely impair visual function or even cause blindness within a short period of time, causing significant distress to patients (6, 7). If the fellow eye of an APAC patient exhibits a shallow anterior chamber, narrow angle, and short axial length, even without elevated IOP, it is considered a primary angle-closure suspect (PACS) under the ISGEO staging system (5).

The pathogenesis of glaucoma remains incompletely understood, with elevated IOP and ocular blood flow dysregulation identified as two major factors in its onset and progression (8–10). Most studies have demonstrated that glaucoma patient’s exhibit compromised perfusion in the optic nerve head and macula area compared to that of normal subjects (11, 12). However, previous research has predominantly focused on primary open-angle glaucoma (POAG), with limited investigation into the microcirculation in PACD. As a distinct subtype of PACD, APAC is of particular clinical significance due to its acute presentation characterized by rapid visual decline and severe visual morbidity. Therefore, in-depth research into APAC is critically important.

Optical coherence tomography angiography (OCTA) is a novel, safe, and non-invasive imaging technique that provides both structural information and quantitative measurements of retinal and choroidal perfusion (13). To date, studies investigating the correlation between glaucoma and vascular factors using OCTA have been largely confined to the 7 mm × 7 mm macular region (14), which represents a relatively limited scanning area. Recent advances in widefield swept-source OCTA (WF SS-OCTA) have enabled comprehensive assessment of retinal perfusion (12 mm × 12 mm region centered on the fovea) in diabebic retinopathy (15), but no studies have applied this technology to PACD. This is the first study to use WF SS-OCTA to evaluate dynamic vascular changes in APAC eyes and the fellow PACS eyes.

Currently, phacoemulsification combined with intraocular lens implantation and gonioscopy-assisted angle surgery has emerged as a safe and effective surgical approach for managing PACD with coexisting cataract (16, 17). In this study, we performed cataract surgery on APAC eyes when IOP was reduced and corneal edema was resolved, as well as on the fellow PACS eyes with coexisting cataract. We quantitatively evaluated changes in macular retinal vascular parameters measured by WF SS-OCTA preoperatively and at 3 months postoperatively in APAC patients. The study aimed to assess whether differences in vascular parameters existed before and after surgery, as well as to investigate the correlation between vascular parameters and structural parameters.

## Methods

### Participants

This retrospective study adhered to the principles of the Declaration of Helsinki and was approved by the Ethics Committee of Shandong Lunan Eye Hospital (Number: 20250401). 31 consecutive patients with one eye of APAC episodes were admitted to the Glaucoma Department of Shandong Lunan Eye Hospital between May 2023 and January 2024. Both APAC eyes and their fellow PACS eyes, totaling 62 eyes were analyzed. The diagnosis of APAC and PACS was based on the ISGEO diagnostic criteria (5). All 31 patients presented with mild to moderate cataracts and underwent bilateral lens extraction combined with intraocular lens implantation. For APAC eyes, gonioscopy-assisted angle surgery was performed concurrently. For all APAC eyes with poorly controlled intraocular pressure (IOP) despite medical therapy, laser peripheral iridoplasty (LPIP) was performed (18). Surgery on APAC eyes was conducted after normalized IOP and corneas becoming clear, with the specific management protocol for acute glaucoma attacks and the detailed surgical procedures are described in the previously published article (19). The postoperative medication regimen were as follows: steroid eye drops (prednisolone acetate 1% QID, tapered over 4 weeks), antibiotic eye drops (gatifloxacin eye drops, QID, for 2 weeks) and NSAID (nepafenac TID for 4 weeks). No systemic anti-inflammatory agents were used. General patient information, including name, sex, age,duration of elevated IOP for APAC eyes (time from symptom onset to initial IOP control),symptom onset to surgery time and systemic conditions, was recorded. In addition, IOP, systolic blood pressure (SBP) and diastolic blood pressure (DBP) were measured at the time of the OCTA imaging. Mean arterial pressure (MAP) was calculated using the following formula: MAP = (SBP + DBP/2)/3. Ocular perfusion pressure (OPP) was calculated by subtracting the IOP from the MAP. All patients underwent baseline ophthalmic examinations at their initial visit, including visual acuity, icare tonometry, slit-lamp biomicroscopy, ocular B-scan ultrasonography, and ultrasound biomicroscopy (UBM). Non-mydriatic fundus photography, corneal endothelial cell examination, and intraocular lens power calculation were performed once intraocular pressure was reduced and corneal edema resolved in APAC eyes. Preoperative WF SS-OCTA (VG200D, SVision Imaging, Luoyang, Henan, China) was performed under mydriasis in both eyes. Postoperative follow-up examinations, including best corrected visual acuity (BCVA), IOP, and slit-lamp biomicroscopy, were conducted at 1 day, 1 week, 1 month, and 3 months. WF SS-OCTA was repeated at 3 months postoperatively.

Inclusion criteria:

1. Age > 40 years;
2. Eyes with a first episode of APAC relieved by medication or laser treatment were included in the APAC group, while fellow eyes without episodes were included in the PACS group;
3. During hospitalization, both APAC and PACS eyes underwent phacoemulsification with intraocular lens implantation, with APAC eyes additionally receiving gonioscopy-assisted angle surgery;
4. APAC eyes presented with a first acute episode, were initially diagnosed at our hospital, had high intraocular pressure lasting less than 1 week, and no history of recurrent subacute episodes;
5. Mild to moderate cataracts were present;
6. Optical media clarity sufficient for OCTA imaging.

Exclusion criteria:

1. Secondary acute angle-closure glaucoma due to causes such as lens intumescence, lens subluxation, or uveitic pupillary block;
2. Previous history of any laser or intraocular surgery [including laser peripheral iridotomy (LPI), laser iridoplasty, trabeculectomy, or other glaucoma-related treatments];
3. History with concurrent intraocular diseases, such as uveitis, diabetic retinopathy, macular degeneration, retinitis pigmentosa, or retinal vascular occlusion;
4. Eyes with opaque optical media or poor imaging quality (OCTA signal strength < 6);
5. Severe dry eye or inability to cooperate with wide field OCTA due to poor patient comprehension;
6. Use of systemic or topical medications affecting ocular blood flow.
7. Intraoperative or postoperative complications (e.g., malignant glaucoma, severe postoperative inflammation, or significant IOP fluctuations) that could potentially affect postoperative blood flow status. Severe postoperative inflammation means anterior chamber cells (>1+) and significant IOP fluctuations points to IOP spikes (>25 mmHg).

### OCTA and OCT imaging

**Figure 1:**
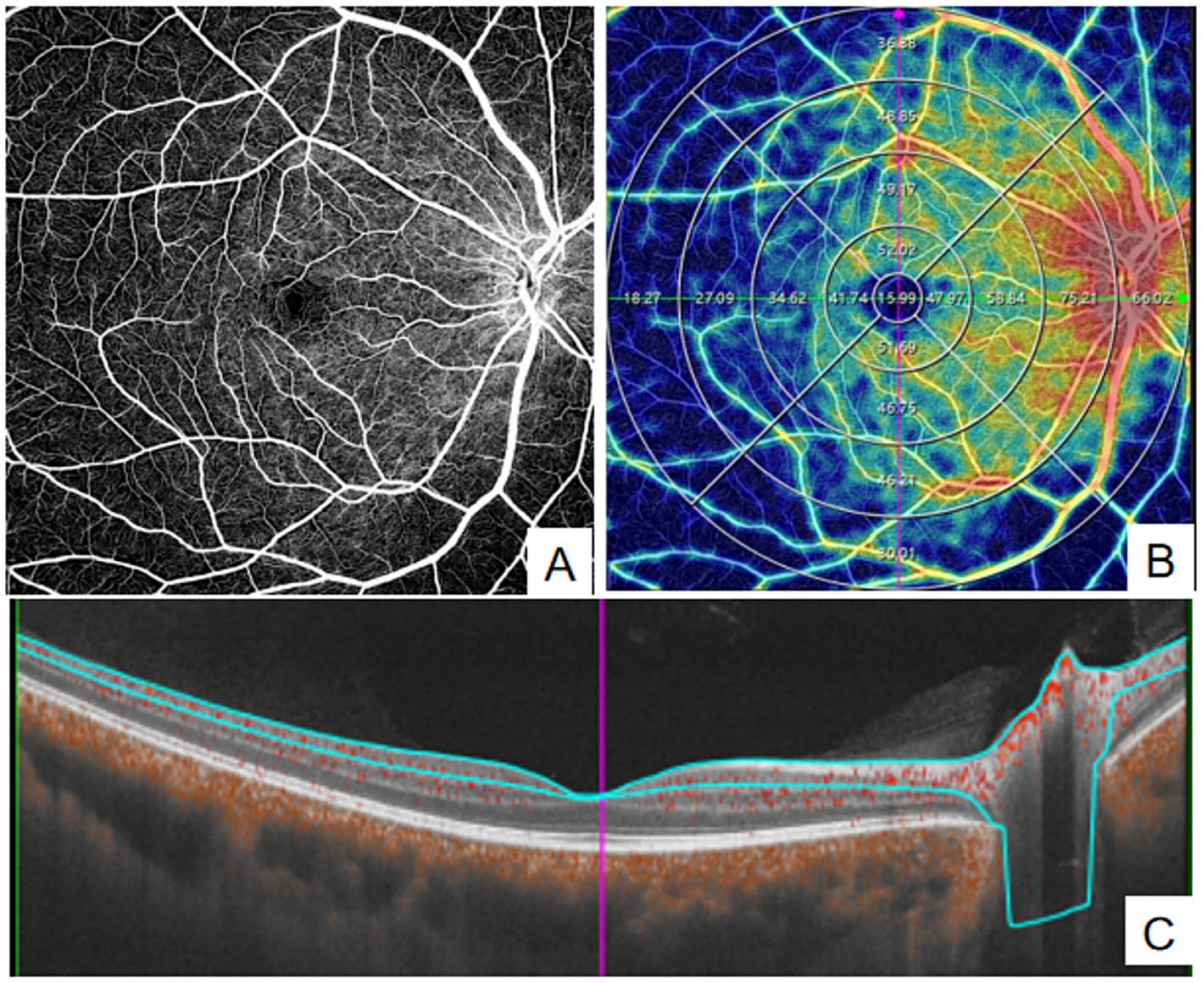
Quantitative measurement of SVC using Wide-field OCTA. **(A)** en face images (12 mm × 12 mm) of color code for vessel density; **(B)** The vessel density of SVC was separately calculated in the concentric rings with different radii (0–3,0-6,0-9,0–12, 3–6,6–9,9-12 mm, and 6–12 mm). **(C)** Structural projection maps of the SVC SVC: superficial vascular complex.

**Figure 2:**
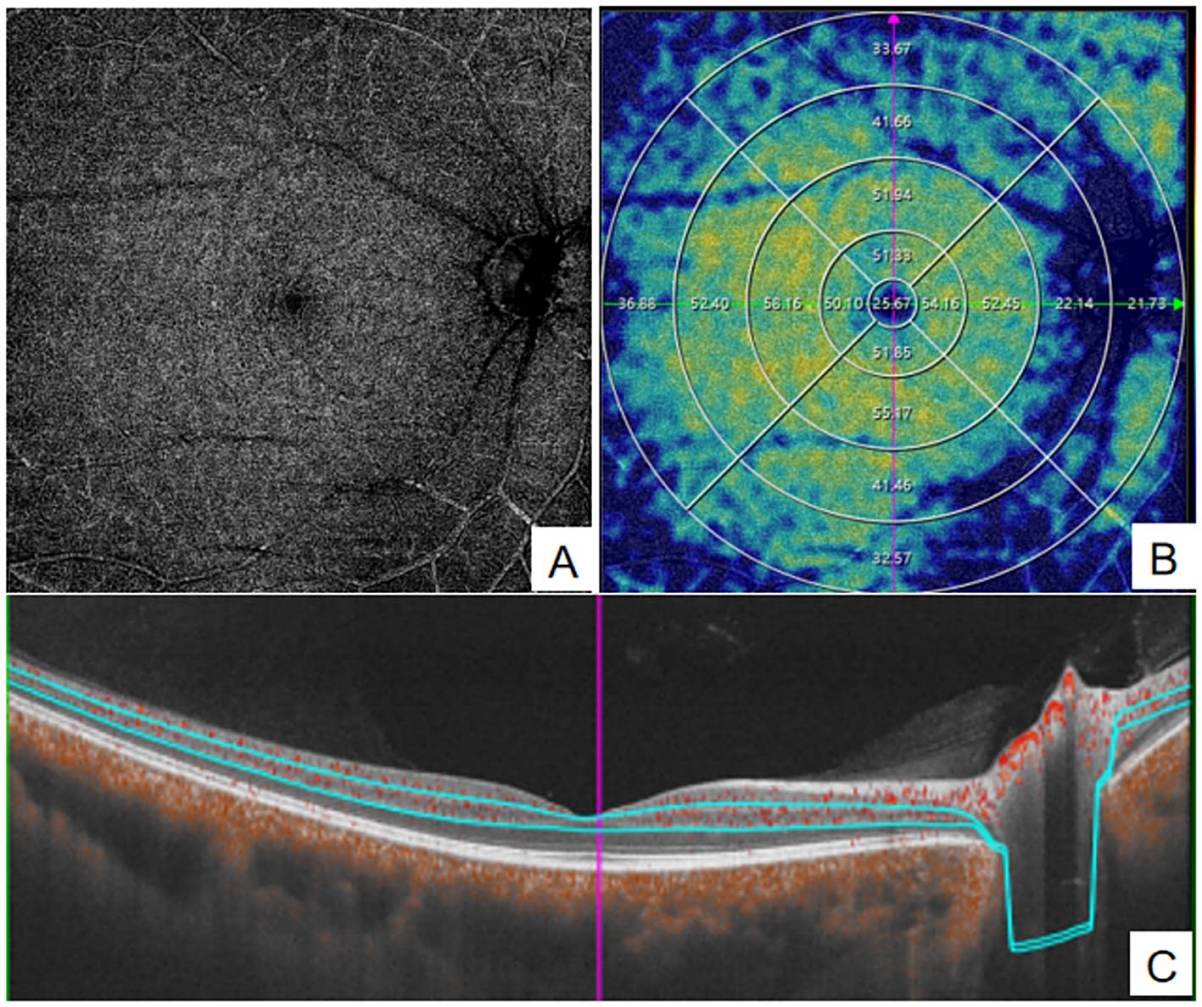
quantitative measurement of DVC using Wide-field OCTA. **(A)** en face images (12 mm × 12 mm) of color code for vessel density; **(B)** The vessel density of DVC was separately calculated in the concentric rings with different radii (0–3,0-6,0-9,0–12, 3–6,6–9,9-12 mm,and 6–12 mm). **(C)** Structural projection maps of the DVC. DVC, deep vascular complex.

OCT and OCTA images were acquired using the VG200D system. The device’s built-in macular “12 mm × 12 mm” model was utilized. OCTA tools were used to generate angiograms of the superficial vascular complex (SVC) and deep vascular complex (DVC). The SVC was defined as the microvasculature extending from 5 μm below the internal limiting membrane (ILM) to the lower one-third of the ganglion cell layer/inner plexiform layer (GCL/IPL) complex (see Figure 1). The DVC was defined as the microvasculature between the lower one-third of the GCL/IPL complex and 25 μm above the outer plexiform layer (OPL) boundary (see Figure 2). Additionally, vessel density (VD) was measured as the percentage (%) of pixels with flow signal above the threshold. The macular mode covered a 12 mm × 12 mm region centered on the fovea. Automatic segmentation of the ganglion cell and inner plexiform layer (GCC) was performed. In the ONH Angio mode, GCC, peripapillary retinal nerve fiber layer (pRNFL), and ONH margin data were obtained from the OCT tools. GCC thickness was measured as the average thickness of the ganglion cell and inner plexiform layers within a 4 × 4.8 mm horizontal ellipse centered on the fovea. A 3.4 mm diameter circle (centered on the optic disc) was automatically placed to measure the average pRNFL thickness and pRNFL thickness in the superior, inferior, nasal, and temporal quadrants. Manual adjustments to the circle position were made if necessary. The built-in software assessed OCTA image quality on a scale from Q1 to Q10. Image quality was automatically scored by the system on a scale of 1 to 10, and images with low grades (quality index < 6) were excluded. All examinations were performed by experienced ophthalmologists.

Parameters measured by OCTA included SVC vessel density, DVC vessel density, GCC thickness, and pRNFL thickness. The macular 12 mm × 12 mm region was divided into four concentric circles (0–3 mm, 3–6 mm, 6–9 mm, and 9–12 mm) using the built-in ETDRS grid for quantitative analysis(see Figures 1, 2). Data for all parameters were obtained and exported using the built-in software of the WF SS-OCTA system.

### Statistical analysis

Statistical analysis was performed using Free Statistics software (version 2.0). Paired T-tests were used to compare preoperative and postoperative parameters between APAC and PACS eyes. Multivariate linear regression analysis was employed to investigate the correlation between macular vascular parameters and structural parameters. Covariates were sequentially added to the base model, and regression coefficients were compared to control for confounding factors. Subgroup analysis was conducted to assess the correlation between vascular parameters and pRNFL thickness. A stratified linear regression model was further applied to analyze subgroups, aiming to evaluate the robustness of the results. Smooth curve fitting was used to illustrate the linear relationship between SVC vessel density in the 0–12 mm macular region and pRNFL thickness.

## Results

The study included 31 patients with a mean age of 62.8 ± 7.2 years, of whom 67.7% were female. Among the participants, 3 with diabetes, 12 with hypertension, the body mass index (BMI) was 24.1 ± 2.9, and the arterial pressure was 97.0 ± 9.8 mmHg. At initial presentation, the intraocular pressure (IOP) in APAC eyes was 50.3 ± 13.8 mmHg, which decreased to 14.6 ± 2.2 mmHg at 3 months postoperatively. In contrast, the IOP in PACS eyes was 16.6 ± 8.3 mmHg at initial presentation and 15.5 ± 3.0 mmHg at 3 months postoperatively. Both the initial and 3-month postoperative visual acuity were significantly better in the PACS group compared to that of the APAC group (see Table 1).

**Table 1:** Baseline characteristics of the participants.

Variables	Total (*n* = 62)	APAC (*n* = 31)	PACS (*n* = 31)	*p*
Sex, *n* (%)				1
Male	–	10 (32.3)	10(32.3)	
Female	–	21 (67.7)	21(67.7)	
Age (y)	–	62.8 ± 7.3	62.8 ± 7.3	1
BMI	–	24.1 ± 2.9	24.1 ± 2.9	1
MAP (mmHg)	–	97.0 ± 9.8	97.0 ± 9.8	1
OPP (mmHg)	82.3 ± 12.3	81.2 ± 14.2	83.4 ± 10.3	0.203
Hypertension, *n* (%)				1
No	–	19 (61.3)	19(61.3)	
Yes	–	12 (38.7)	12(38.7)	
Dibates, *n* (%)				1
No	–	28 (90.3)	28(90.3)	
Yes	–	3 (9.7)	3(9.7)	
EYE				0.075
OD	31 (50.0)	19 (61.3)	12(38.7)	
OS	31 (50.0)	12 (38.7)	19(61.3)	
Duration (d)	1.6 ± 2.2	3.2 ± 2.1	–	–
Symptom onset to surgery time	4.0 ± 7.3	7.9 ± 8.8	–	–
BCVA-first visit (logMAR)				<0.001
≤0.01	8 (12.9)	0 (0)	8(25.8)	
>0.1,≤0.3	15 (24.2)	3 (9.7)	12(38.7)	
>0.3,≤1.0	21 (33.9)	12 (38.7)	9(29)	
>1.0	18 (29.0)	16 (51.6)	2(6.5)	
BCVA-postoperation (logMAR)				0.041
≤0.01	26 (41.9)	8 (25.8)	18(58.1)	
>0.1,≤0.3	28 (45.2)	18 (58.1)	10(32.3)	
>0.3,≤1.0	7 (11.3)	4 (12.9)	3(9.7)	
>1.0	1 (1.6)	1 (3.2)	0(0)	
IOP-first visit (mmHg)	33.5 ± 20.4	50.3 ± 13.8	16.6 ± 8.3	<0.001
IOP-postoperation (mmHg)	15.1 ± 2.6	14.6 ± 2.2	15.5 ± 3.0	0.11
ACD (mm)	1.7 ± 0.3	1.57 ± 0.27	1.74 ± 0.29	0.013
LT (mm)	5.0 ± 0.3	5.00 ± 0.24	4.96 ± 0.27	0.278
AL (mm)	22.3 ± 0.5	22.25 ± 0.54	22.32 ± 0.45	0.337
CCT (μm)	547.1 ± 41.0	554.65 ± 8.48	539.58 ± 5.88	0.03

MAP, mean arterial pressure; OPP, ocular perfusion pressure; Duration, means duration of elevated IOP for APAC eyes; BCVA, best corrected visual acuity; BCVA1, visual acuity at Presentation; BCVA2, visual acuity at 3 Months postoperatively; IOP, intraocular pressure; IOP1, IOP at presentation; IOP2, IOP at 3 Months postoperatively; ACD, Anterior Chamber Depth; LT, lens thickness; AL, axial length; CCT, central corneal thickness.

In the PACS group, there were no statistically significant differences in vascular density in a 12 mm × 12 mm region centered on the fovea and structural parameters before and after surgery (*p* > 0.05). In the APAC group, compared to preoperative values, the SVC vessel density in the 0–6 mm region decreased,but the difference was not statistically significant postoperatively(*p* > 0.05), while significant reduction was observed in the 6–9 mm and 9–12 mm concentric annular regions, with statistical significance (*p* < 0.05). In contrast, the DVC vessel density increased significantly across all measuring regions postoperatively, with statistical significance (*p* < 0.05). Analysis of structural parameters in the optic disc region of the APAC group revealed that, compared to preoperative values, the average pRNFL thickness, pRNFL thickness in all quadrants, and average GCC thickness were significantly reduced postoperatively, with statistical significance (*p* < 0.05, see Table 2).

**Table 2:** Changes in Blood Flow Parameters, RNFL Thickness, and GCC Thickness Before and After Surgery in the PACS and APAC Groups.

Variables	Region (mm)	PACS			APAC		
		Preoperative (*n* = 31)	Postoperative (*n* = 31)	*p*	Preoperative (*n* = 31)	Postoperative (*n* = 31)	*p*
SVC	0–6	40.3 ± 6.3	41.7 ± 6.7	0.237	37.1 ± 7.2	37.5 ± 8.8	0.818
	0–12	41.0 ± 4.8	41.2 ± 5.1	0.778	39.1 ± 4.9	36.3 ± 6.7	0.012
	6–12	41.2 ± 4.9	41.0 ± 5.0	0.794	39.8 ± 4.7	35.9 ± 6.8	0.001
	6–9	46.4 ± 6.2	46.6 ± 5.7	0.726	45.1 ± 6.1	40.8 ± 8.2	0.04
	9–12	37.4 ± 5.4	36.9 ± 6.1	0.421	35.9 ± 5.1	32.3 ± 6.4	<0.001
DVC	0–6	49.5 ± 7.2	50.7 ± 6.8	0.303	43.3 ± 6.7	48.9 ± 9.4	0.014
	0–12	34.5 ± 5.7	34.2 ± 4.9	0.602	28.8 ± 4.4	32.1 ± 6.3	0.019
	6–12	29.4 ± 6.2	28.6 ± 5.3	0.306	23.1 ± 3.7	26.5 ± 6.3	0.01
	6–9	33.1 ± 7.7	33.8 ± 6.1	0.645	27.9 ± 5.3	31.9 ± 7.6	0.014
	9–12	26.8 ± 5.9	24.9 ± 6.2	0.079	19.2 ± 3.3	22.5 ± 6.6	0.013
P-RNFLT	S	141.0 ± 22.0	138.6 ± 22.5	0.539	155.9 ± 47.6	114.8 ± 34.1	<0.001
	I	150.1 ± 14.4	146.6 ± 19.3	0.225	163.7 ± 43.0	128.5 ± 35.1	<0.001
	N	89.4 ± 14.7	89.1 ± 16.1	0.921	99.9 ± 33.3	79.5 ± 19.3	<0.001
	T	86.1 ± 10.5	85.8 ± 11.3	0.809	91.3 ± 15.8	80.9 ± 16.6	<0.001
	Average	116.7 ± 11.1	115.0 ± 12.4	0.466	127.7 ± 32.1	100.9 ± 23.1	<0.001
GCC-T		80.0 ± 7.1	80.2 ± 7.0	0.059	75.5 ± 10.6	75.4 ± 10.7	0.386

SVC, superficial vascular complex; DVC, deep vascular complex; P-RNFLT, peripapillary retinal nerve fiber layer thickness; S, superior; I, inferior; N, nasal; T, temporal; GCC-T, ganglion cell complex thickness.

**Figure 3:**
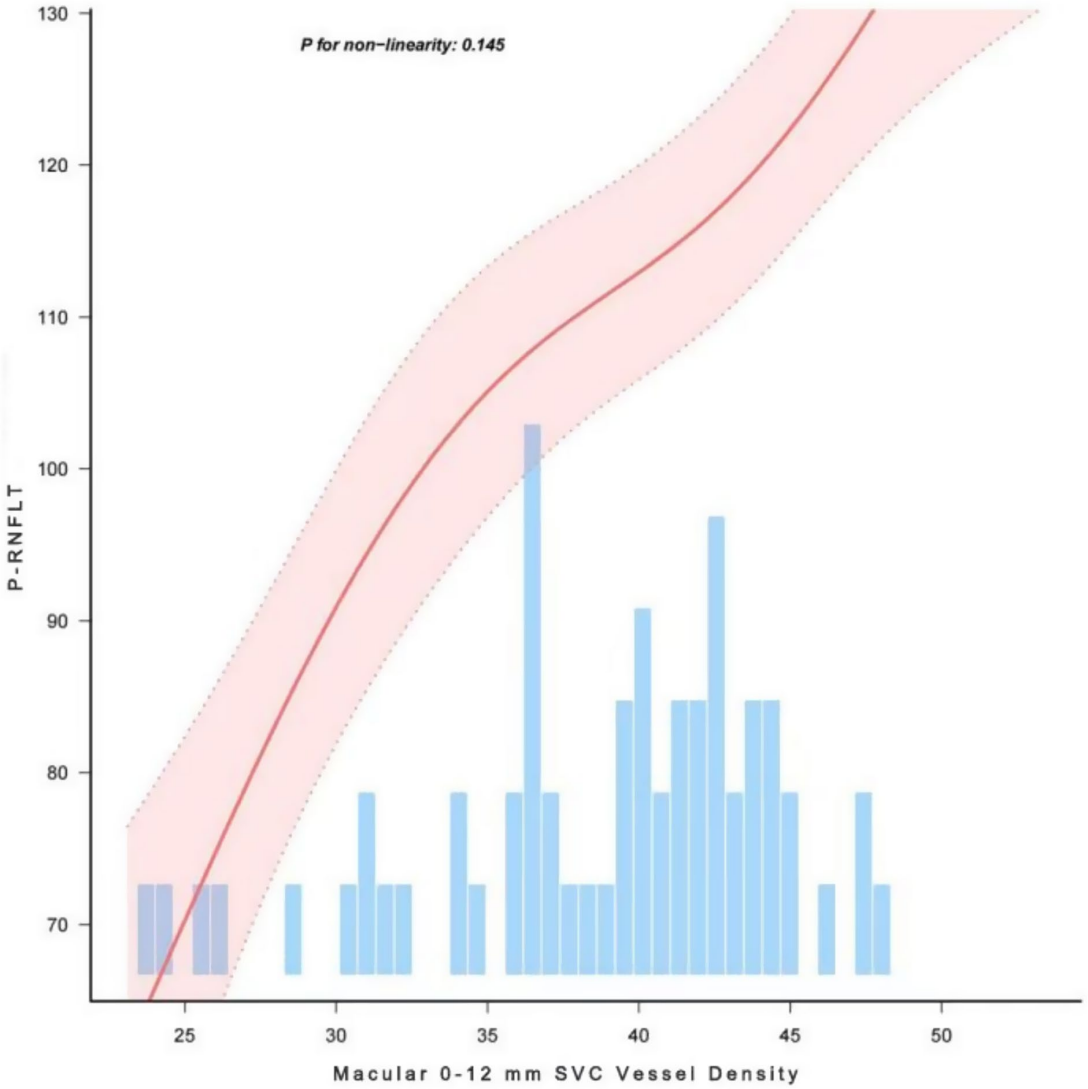
Correlation between Macular 0–12 mm SVC Vessel Density and pRNFL Thickness.

The study demonstrated that GCC thickness, as well as SVC vessel density in the 0–6 mm, 0–12 mm, and 6–12 mm regions, were positively correlated with pRNFL thickness (*p* < 0.001). Table 3 presents the *β* values and 95% confidence intervals (CI) for the three models. In Model I, after adjusting for age, sex, BMI, hypertension status, and diabetes status, the results showed that a 1% decrease in SVC vessel density in the 0–12 mm region was associated with a 2.42 μm decrease in pRNFL thickness, while a 1 μm decrease in GCC thickness was associated with a 1.38 μm decrease in pRNFL thickness. In Model II, which further adjusted for eye laterality, baseline visual acuity, baseline intraocular pressure, and duration of elevated intraocular pressure, a 1% decrease in SVC vessel density in the 0–12 mm region was associated with a 2.48 μm decrease in pRNFL thickness, and a 1 μm decrease in GCC thickness was associated with a 1.23 μm decrease in pRNFL thickness. Additionally, smooth curve fitting was used to demonstrate a positive linear relationship between SVC vessel density in the 0–12 mm region and pRNFL thickness (nonlinear *p* > 0.05; Figure 3), which is consistent with the results presented in Table 3.

**Table 3:** Correlation analysis between various parameters and average peripapillary retinal nerve fiber layer thickness.

Variables	Region(mm)	Non-adjusted β (95% CI)	*p*	Model I β(95% CI)	*p*	ModelI I β (95% CI)	*p*
SVC	0–6	1.68 (1.22,2.14)	<0.001	1.69 (1.18 ~ 2.2)	<0.001	1.47 (0.92 ~ 2.02)	<0.001
	0–12	2.46 (1.99,2.93)	<0.001	2.42 (1.77 ~ 3.07)	<0.001	2.48 (1.95 ~ 3.01)	<0.001
	6–12	2.37 (1.87,2.86)	<0.001	2.26 (1.7 ~ 2.81)	<0.001	2.16 (1.49 ~ 2.83)	<0.001
GCC-T		1.51 (1.12,1.9)	<0.001	1.38 (0.94 ~ 1.82)	<0.001	1.23 (0.78 ~ 1.68)	<0.001

SVC, superficial vascular complex; GCC-T, ganglion cell complex thickness. Model I: Adjusted for sex, age, and BMI; Model II: Further adjusted for diagnosis, hypertension status, and diabetes status in addition to Model I.

**Figure 4:**
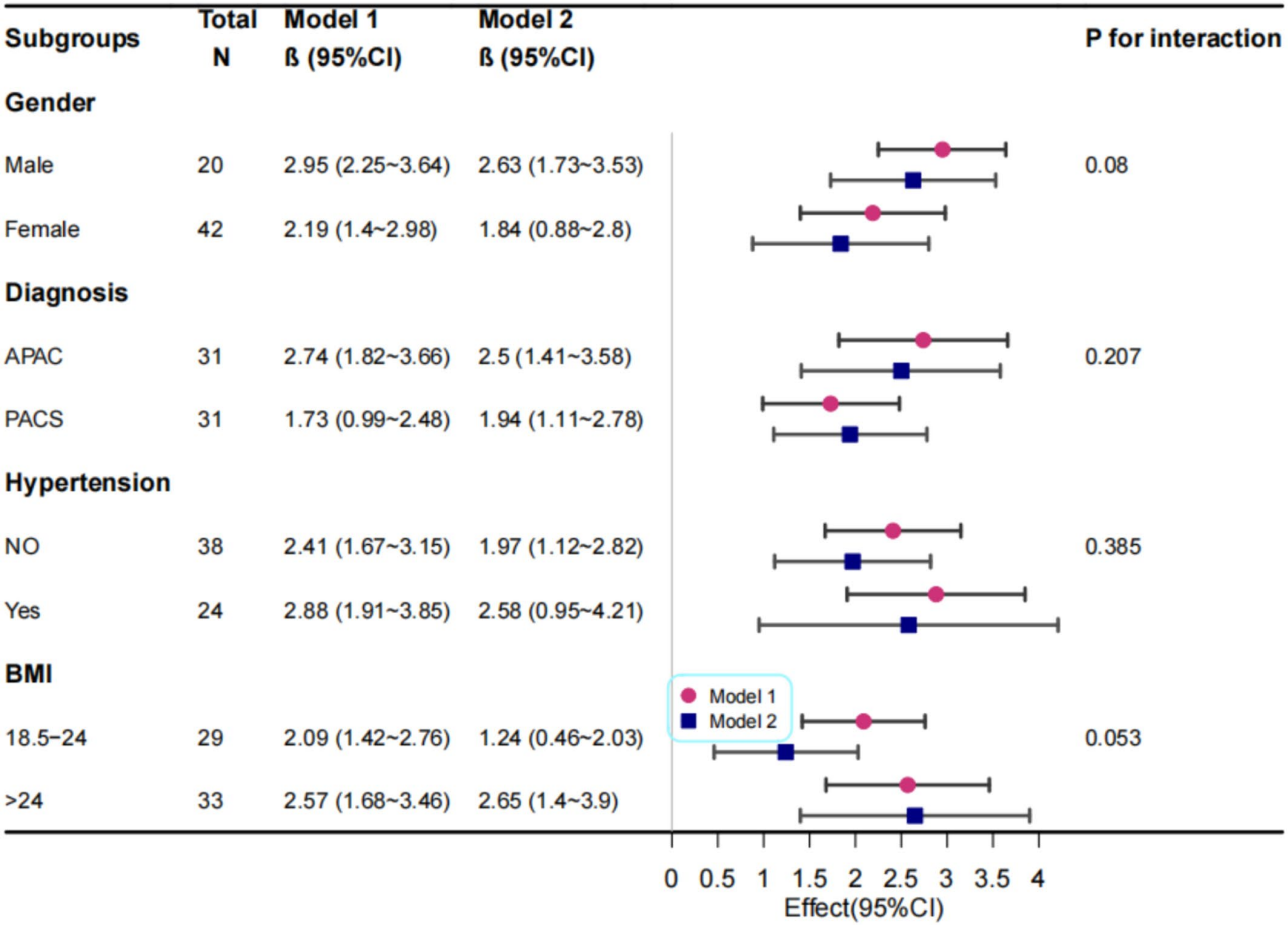
Correlation Analysis Between Macular 0-12 mm SVC and RNFL Thickness. Model I: Adjusted for sex, age, and BMI; Model II: Further adjusted for diagnosis, hypertension status, and diabetes status in addition to Model I variables.

We further conducted stratified analyses to evaluate the robustness of the correlation between SVC vessel density in the 0–12 mm region and pRNFL thickness across various subgroups (sex, BMI, glaucoma type, and hypertension status) (Figure 4). No significant interactions were observed in any of the subgroups (all interaction p > 0.05), indicating that the correlation between SVC vessel density in the 0–12 mm region and pRNFL thickness remained consistent and robust across different subgroups.

## Discussion

Most previous studies utilized OCTA devices with limited scanning ranges, typically confined to the area in a 7 mm × 7 mm region centered on the fovea (14). Due to these spatial constraints, important information outside the scanned area may have been missed. In this study, by examining the inner retinal vessel density in a 12 mm × 12 mm region centered on the fovea, ocular blood flow perfusion were unable to be assessed more comprehensively.

In the PACS group, no statistically significant differences were observed in any parameters before and after surgery, suggesting that the impact of phacoemulsification on vessel density had recorved by 3 months postoperatively. This finding aligns with previous studies reporting no significant changes in peripapillary retinal vessel density at 3 months following cataract surgery (20). Since the surgical approach and cataract severity (mild to moderate) were consistent between the APAC and PACS groups in our study, the influence of cataract surgery itself on blood flow perfusion could be largely ruled out.

Previous studies have shown that the SVC density in the macular area of APAC patients is significantly affected at 3 months postoperatively (21, 22). In our study, although SVC density in the 0–6 mm region of APAC eyes showed a non-significant decrease at 3 months postoperatively, a significant reduction was observed in the 6–12 mm annular region. This suggests that even with stable intraocular pressure (IOP), reduction in superficial retinal vessel density persists, particularly in regions farther from the fovea. This regional variability underscores the clinical value of wide field OCTA.

Basic research has demonstrated that elevated IOP induces sustained endothelial dysfunction and impaired autoregulation of retinal arterioles, which persist irreversibly even after IOP normalization (23). Pericyte loss, potentially resulting from increased oxidative stress due to elevated IOP, may further disrupt endothelial function (24, 25). Thus, we hypothesize that the irreversible endothelial damage and impaired autoregulation caused by high IOP continue to persists even after IOP stabilization.

The improvement in DVC density across all regions may be attributed to functional hyperemia (26). This theory posits that restored light stimulation following cataract extraction increases neuronal activity, leading to the release of vasodilatory metabolites such as adenosine, lactate, and arachidonic acid, which induce functional hyperemia to meet the heightened energy demands of ganglion cells (27, 28). In our study, all patients underwent phacoemulsification for mild to moderate cataracts, with short surgical duration. By 3 months postoperatively, the effects of phacoemulsification on retinal perfusion had resolved (20). Therefore, the improvement of vessel density in macular area should be due to enhanced light stimulation following cataract removal, which increases neuronal activity and induces vasodilation. Notably, stronger light stimuli or prolonged dark adaptation can lead to more pronounced increases in blood flow (29). In APAC patients, corneal edema during episodes of elevated IOP leads to significantly reduced visual acuity and diminished light stimulation. Compared to PACS patients, APAC patients, who experience a period of dark adaptation, exhibit persistent pupillary dilation that does not resolve postoperatively, resulting in increased light entry into the eye. Consequently, APAC eyes receive greater light stimulation compared to that of PACS eyes, which may explain the more pronounced improvement in DVC perfusion postoperatively. This suggests that cataract extraction in APAC patients enhances light stimulation, potentially improving their blood flow perfusion status (30).

However, despite increased light stimulation postoperatively, SVC density in APAC eyes continued to decline at 3 months, indicating that elevated IOP primarily damages superficial vascular cell function, and this damage persists even after IOP stabilization. This vascular impairment, leading to reduced perfusion, cannot be fully compensated by increased neuronal activity. Therefore, it is crucial to normalize IOP in APAC patients promptly to prevent further endothelial damage. Our findings advocate for integrating WF SS-OCTA into routine APAC follow-up protocols. Monitoring SVC density in the 0–12 mm region may serve as an early biomarker for irreversible glaucomatous damage, guiding timely intervention.

To explore the correlation between RNFL thickness and GCC thickness, as well as the relationship between blood flow perfusion and structural changes, we investigated the associations between peripapillary RNFL thickness and both macular GCC thickness and SVC vessel density. Given potential measurement errors due to retinal edema during acute IOP elevation, we focused on postoperative correlations at 3 months. Our findings revealed positive correlations between peripapillary RNFL thickness and both macular GCC thickness and SVC vessel density, consistent with previous studies linking retinal vessel density to GCC thickness (31). In APAC eyes, even with stable IOP, reductions in SVC density were associated with RNFL thinning. This aligns with a retrospective study showing that, despite stable IOP, one-third of Asian APAC patients developed severe optic disc cupping (>0.9) over 6 years, with over 90% maintaining controlled IOP (32). Our results provide a plausible explanation for this phenomenon.

This study has several limitations. First, it is a single-center study with a relatively small sample size. Second, the follow-up period was limited to 3 months after APAC attack; longer follow-up is needed to evaluate changes in blood flow perfusion over time and longitudinal studies are needed to validate whether SVC density predicts long-term visual field loss. Additionally, our findings are specific to investigate the APAC patients with acute IOP elevation lasting less than 1 week and may not generalize to all APAC cases.

In conclusion, our study demonstrates that, at 3 months after APAC attack, APAC eyes exhibit reductions in SVC density in the 6–12 mm annular region centered on the fovea despite stable IOP, while DVC density shows partial recovery in a 12 mm × 12 mm region centered on the fovea. Positive correlations between peripapillary RNFL thickness and both GCC thickness and macular SVC vessel density suggest underlying axonal loss with concomitant microvascular compromise in APAC eyes. Clinically, close monitoring of SVC density and RNFL thickness is essential in these patients. Compared to conventional OCTA, widefield OCTA provides more comprehensive blood flow information and holds significant promise for future applications.

## Data Availability

The raw data supporting the conclusions of this article will be made available by the authors, without undue reservation.
